# New Literature

**DOI:** 10.1155/S146392468100014X

**Published:** 1981


### New Literature

### AA spectrophotometer

The Model 4000 atomic absorption spectrophotometer is featured in a brochure from Perkin-Elmer. Instrument, data handling capabilities, optical performance and many accessories are fully described and illustrated.
*Perkin-Elmer Ltd, Post Office Lane, Beaconsfield, Bucks, UK.*

### Analysis of coal and coke

A brochure from Fisher Scientific introduces their coal analyser. It is claimed that this is the only instrument on the market with the capability to analyse a single sample for % moisture, volatiles, ash and fixed carbon. Although designed for the coal industry, it can also be used to determine moisture and volatiles in other products such as chemicals, foods, pharmaceuticals and paper. Microprocessor control totally automates the heat programs, atmosphere control and all calculations. Its results meet or exceed reproducibility and repeatability requirements of ASTM method No. D3172.
*Fisher Scientific Co, 711 Forbes Ave, Pittsburgh, PA 15219, USA.*

### Ion chromatography system

An application note from Techmation describes the use of the Wescan Model 260 ion chromatography system with conductometric detection for the measurement of alkyl sodium sulphates.
*Techmation Ltd, 58 Edgeware Way, Edgeware, Middx. HA8 8JP, JK.*

### Multi-demensional GC

Packard have produced a brochure on the applications of multi-dimensional gas chromatography. Information includes the theory of multi-dimensional GC, a number of important applications and the operation and configurations of the system.
*Packard Instrument Ltd, 13-17 Church Rd, Caversham, Berks. RG4 7AA, UK.*

### Negative ion GC/MS

An application report entitled "Biomedical Applications of Negative Ion Chemical Ionization Mass Spectrometry" is available from Finnigan Corp. The paper shows the use of negative ion spectra as an ancillary technique to corroborate structural assignments (unambiguous molecular weight determination) from electron impact and positive ion chemical ionisation.
*Finnigan Corp., 845 West Maude Ave., Sunnyvale, CAL. 94086, USA.*

### HPLC of aflatoxins

The use of fluorescence detection in the HPLC analysis of aflatoxins is discussed in a report published by the Schoeffel Instrument Division of Kratos. The method presented in this report establishes a routine procedure for the separation and quantitative detection of aflatoxins $B_1$, $B_2$, $G_1$, and $G_2$ at picogram levels.
*Kratos Inc, Schoeffel Instrument Div., 24 Booker St., Westwood, NJ 07675, USA.*

### Automated analysis of natural gas

"An automated method for the analysis of natural gas and light petroleum" is the title of a paper published in Vol. 8 No. 1 of Perkin-Elmer's "Chromatography Newsletters".
*Perkin-Elmer Ltd, Post Office Lane, Beaconsfield, Bucks, HP9 1QA, UK.*

### Synthetic fuels

The American Institute of Chemical Engineers has published a booklet entitled "Synthetic Fuels" that explains the potential of replacing oil imports with synthetic fuels from coal and oil shale.
*AIChE, Public Communications Dep., 345 E. 37th St., New York, NY 10017, USA.*

### Liquid scintillation

Packard have published detailed brochures on their Tri-Carb 300C and 460C automatic liquid scintillation systems. Both systems incorporate the Spectralyzer which computes the sample count ratio, spectral index of the sample and spectral index of the external standard.
*Packard Instrument International SA, Renggerstrasse 3, CH-8038 Zurich, Switzerland.*

### Fluorescence detection in LC

A new book entitled "Fluorescence detection in liquid chromatography" by A.T. Rhys-Williams has been published by Perkin-Elmer. The book is available direct from Perkin-Elmer at £3.95 inclusive of postage and packing. The object of the book is to familiarise chemists to whom fluorescence is a new technique with the basic concepts.
*Perkin-Elmer Ltd, Post Office Lane, Beaconsfield, Bucks, HP9 1QA, UK.*

### Fast protein analyser

A brochure and several application reports on their P-100 protein analyser have been published by Newport Instruments. The P-100 is a microprocessor-based NMR spectrometer designed for use in the food processing industry and wherever there is a requirement to check protein percentages frequently.
*Newport Instruments Ltd, Blakelands North, Milton Keynes, MK14 5AW, UK.*

### Ion chromatograms

Dionex are distributing from their UK office a data sheet describing some applications of their ion chromatography system. Fourteen chromatograms including anion and cation analysis of effluents, fuels and low level determination of perchlorate are given.
*Dionex (UK) Ltd, The Parade, Frimley, Camberley, Surrey, GU16 5HY, UK.*

### Accessories for automated analysis

"Laboratory products for automated chemistry" is the title of a booklet from Sterelin Instruments. In addition to describing their range of glassware and accessories for automatic analysis, the booklet includes information on the FP.9 discrete analyser system.
*Sterelin Instruments, Mill Lane Estate, Alton, Hants, GU34 2PL, UK.*

### Infra-red process analyser

An illustrated brochure from Foxboro Analytical describes the expanded capability of the Miran II process analyser in high-speed industrial applications where the ability to detect and react instantaneously to specific concentrations of one or more compounds in a process stream is an increasing requirement.
*Foxboro Analytical, 28 Heathfield, Stacey Bushes, Milton Keynes, Bucks, MK12 6HR, UK.*

### Automated analysis

Technicon Industrial Systems has published a special issue of the Environmental Sciences Newsletter, a publication dealing with developments in automated analysis. The current issue contains information on EPA-proposed changes to approved automated test procedures, and an interview with Robert Booth, Deputy Director of the Environmental Monitoring and Support Laboratory, who discusses proposed changes to methods for total phosphorus, TKN, sulphate, COD, cyanide and phenolics. Included are articles on use of automated continuous-flow systems at three environmental laboratories: Baltimore Wastewater Laboratories where automated systems are used for accumulation of nutrient data and control of wastewater treatment plants; the US Army Environmental Hygiene Agency, where four analysers installed in a mobile van enable water quality studies to be speeded up; and the Occoquan Laboratory, Virginia, where automated systems are used for characterisation of stormwater runoff to the Occoquan Watershed, for support of the Nationwide Urban Runoff Project and for monitoring drinking water for suburbs of Washington, DC.
*Technicon Industrial Systems, Tarrytown, New York 10591, USA.*

